# Structural and mechanistic basis for the regulation of the chloroplast signal recognition particle by (p)ppGpp

**DOI:** 10.1002/1873-3468.70008

**Published:** 2025-02-11

**Authors:** Victor Zegarra, Paul Weiland, Pauline Anka Plitzko, Julia Thiery, Laura Czech, Felix Willmund, Patricia Bedrunka, Gert Bange

**Affiliations:** ^1^ Center for Synthetic Microbiology (SYNMIKRO) Philipps University Marburg Germany; ^2^ Department of Chemistry Philipps‐University Marburg Germany; ^3^ Center for Tumor Biology and Immunology, Department of Medicine Philipps‐University Marburg Germany; ^4^ Department of Biology Philipps‐University Marburg Germany; ^5^ Max Planck Institute for Terrestrial Microbiology Marburg Germany

**Keywords:** (p)ppGpp, alarmones, chloroplast, nucleotide second messenger, plants, signal recognition particle

## Abstract

The alarmones (p)ppGpp play a critical role in chloroplasts by acting as signalling molecules that regulate gene expression, protein synthesis and chloroplast (*cp*) development, particularly in response to stress and nutrient availability. However, the underlying molecular mechanisms are still poorly understood. Here, we show that (p)ppGpp binds to the GTPase‐containing NG domains of the chloroplast signal recognition particle (SRP) and its receptor, preventing their GTP‐dependent association through a competitive mechanism. The structure of (*cp*)FtsY bound to ppGpp reveals that the alarmone employs the same binding mode as its GDP counterpart and hinders chloroplast SRP:FtsY complex formation via its pyrophosphate moiety. Consequently, (p)ppGpp also inhibits the mutual stimulation of the two GTPases present in the (*cp*)SRP54:FtsY complex. Taken together, our findings provide the first description of how the alarmones (p)ppGpp may regulate the SRP‐dependent protein trafficking pathway in plants.

## Graphical abstract

LHCPs are transported to the thylakoid membrane via the (*cp*)SRP pathway. This process involves a transit complex of (*cp*)SRP43, (*cp*)SRP54 and LHCP, which interacts with (*cp*)FtsY and Alb3 at the membrane. GTP hydrolysis by (*cp*)SRP54 and (*cp*)FtsY triggers complex dissociation. This study shows that the alarmones (p)ppGpp regulate this pathway by binding to (*cp*)SRP54 and (*cp*)FtsY, inhibiting their interaction and GTPase activity.

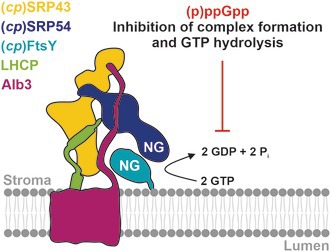

## Abbreviations

**cp**, chloroplast

**SRP**, signal recognition particle

**(*cp*)SRP**, chloroplast signal recognition particle

**GDP**, guanosine diphosphate

**GTP**, guanosine triphosphate

**RSH**, RelA/SpoT homologue

**ppGpp**, guanosine tetraphosphate

**pppGpp**, guanosine pentaphosphate

The signal recognition particle (SRP) is a ubiquitous cellular complex responsible for the precise targeting of secretory and membrane proteins to their corresponding destinations [1, 2]. The core components of the cytosolic SRP, namely the SRP‐RNA and SRP54 (also named Ffh in bacteria), coordinate the recognition of signal sequences emerging from translating ribosomes and the delivery of an SRP:ribosome‐nascent chain complex to the membrane‐bound SRP receptor, FtsY. This process also occurs post‐translationally, whereby the SRP recognizes signal sequences once translation has been completed [3].

The chloroplast (*cp*) functions as the hub of photosynthesis in plant cells, relying on highly regulated mechanisms to ensure precise localization of membrane proteins and preserve the structural integrity of the thylakoid membrane. Among these mechanisms, the chloroplast (*cp*)SRP of higher plants exhibits unique structural and functional characteristics in comparison with its well‐studied cytosolic counterpart, notably lacking the RNA constituent that accelerates complex formation between the SRP and SRP receptor GTPases [4, 5, 6, 7]. The key components of the (*cp*)SRP include (*cp*)SRP54 and (*cp*)SRP43, which form a high‐affinity complex [8, 9, 10]. The (*cp*)SRP54:SRP43 complex interacts with conserved motifs of proteins, which were post‐translationally imported from the plant cytoplasm into the chloroplast, and delivers them to the thylakoid membrane [9, 11]. Additionally, (*cp*)SRP43 acts as a chaperone for light‐harvesting complex proteins (LHCPs) [12]. The chloroplast SRP receptor (*cp*)FtsY interacts with (*cp*)SRP54 to facilitate the transfer of protein cargo to the Alb3 insertase in a GTP‐dependent manner [13, 14]. Kinetic data showed that (*cp*)SRP54 and (*cp*)FtsY can interact efficiently with each other, thereby obviating the need for an SRP‐RNA [15].

The bacterial stringent response is a stress‐induced signalling mechanism accompanied by the accumulation of the alarmones ppGpp and pppGpp, which are derivatives of GDP and GTP, respectively, carrying a pyrophosphate at the 3′‐OH group of their ribose moieties. These alarmones (collectively also referred to as (p)ppGpp) drive a global reprogramming of metabolism and gene expression to adapt to the stress condition (reviewed in: [16]). The synthesis of (p)ppGpp is catalysed by RelA/SpoT homologue (RSH) proteins [17, 18, 19, 20] and regulates various target proteins, including RNA polymerase and translational GTPases [16]. This process is tightly controlled, with (p)ppGpp synthesis and hydrolysis being modulated in response to environmental conditions [21, 22, 23, 24, 25].

Although the stringent response has been extensively studied in bacteria, several studies have shown that the (p)ppGpp‐mediated regulation of cellular processes is also conserved in the chloroplasts of algae and plants (reviewed in: [26, 27]). Plants possess nuclear‐encoded RSH proteins localized in chloroplasts, and ppGpp has been detected within these organelles [28, 29, 30]. Increased levels of ppGpp were observed during night periods and under transient dark treatments, reaching a concentration of approximately 3 μm [30]. Beyond its role in diurnal rhythms, ppGpp also plays a key role in chloroplast adaptation to nitrogen deprivation by adjusting the photosynthetic electron transport chain to supress photosynthetic activity [31]. Additionally, increased levels of ppGpp seem to influence plant growth and development [32, 33, 34]. Emerging evidence also highlights its role in plant immunity, where an overaccumulation of the alarmone has been associated with reduced expression of defence‐related genes and greater susceptibility to pathogens, while lower ppGpp levels correlate with increased resistance and enhanced salicylic acid accumulation [35, 36]. To date, only the plastidial RNA polymerase from *Spinacia oleracea*, the adenylosuccinate synthetases from *Oryza sativa* and the guanylate kinase from *Oryza sativa*, *Pisum sativum* and *Arabidopsis thaliana* have been identified as direct binders of ppGpp in plants [37, 38, 39]. However, the precise mechanism of action of (p)ppGpp in chloroplasts remains elusive.

In bacteria, (p)ppGpp binds to the proteins Ffh and FtsY, interfering with their GTP‐dependent interaction. This disruption hinders the SRP‐dependent targeting of membrane proteins [40]. Given that chloroplasts harbour homologues of several components of the bacterial SRP system, we hypothesized that (p)ppGpp might similarly regulate the (*cp*)SRP system. In the present work, we present biochemical and structural data confirming the binding of (p)ppGpp to (*cp*)SRP54 and the NG domain of (*cp*)FtsY (referred to as (*cp*)FtsY‐NG henceforth). Our findings demonstrate that this interaction disrupts the complex formation between the two GTPases, resulting in a significant reduction in GTP hydrolysis, a crucial step for membrane protein insertion. Our findings not only identify the chloroplast SRP as a target of (p)ppGpp but also broaden our understanding of the conservation of (p)ppGpp signalling and its potential implications in plant stress responses and plastid function.

## Materials and methods

### Plasmid construction, gene expression and protein purification

Gene fragments encoding residues 65–366 of *Arabidopsis thaliana* (*cp*)FtsY‐NG (UniProt: O80842) and residues 79–564 of *Arabidopsis thaliana* (*cp*)SRP54 (UniProt: P37107) were cloned as previously described [41]. The corresponding hexa‐histidine tag fusion proteins were produced in *Escherichia coli* BL21 (DE3) Rosetta using three litres of LB medium supplemented with 1% (w/v) lactose and 50 μg·mL^−1^ kanamycin for 16 h at 30 °C. Cells were harvested by centrifugation (5000 ***g*** for 10 min, 4 °C) and lysed in Buffer A (20 mm HEPES‐K, pH 8.0; 250 mm NaCl; 20 mm KCl and 50 mm imidazole) using a microfluidizer at 12 000 psi (M‐110 L, Microfluidics; Westwood, MA, USA). The resulting lysate was then clarified by centrifugation (20 000 ***g*** for 20 min, 4 °C) and the supernatant was loaded into a 1 mL HisTrap FF column (Cytiva; Marlborough, MA, USA). Proteins were eluted in Buffer B (20 mm HEPES‐K, pH 8.0; 250 mm NaCl; 20 mm KCl and 250 mm imidazole). To promote the removal of potentially bound nucleotides, (*cp*)SRP54 and (*cp*)FtsY‐NG went through an additional 20‐min incubation with 30 mm EDTA at room temperature prior to being concentrated in an Amicon Ultra Centrifugal Filter, 10 kDa MWCO (Millipore; Darmstadt, Hessen, Germany). All proteins then went through a second round of purification by size‐exclusion chromatography in a HiLoad 16/60 Superdex 200 prep grade column (Cytiva; Marlborough, MA, USA) in buffer C (20 mm HEPES‐K, pH 7.5; 200 mm NaCl; 20 mm KCl and 20 mm MgCl_2_), and the respective protein‐containing fractions were then concentrated in an Amicon Ultra Centrifugal Filter, 10 kDa MWCO (Millipore). Protein concentrations were determined using a NanoDrop Lite Spectrophotometer (Thermo Fisher Scientific; Schwerte, NRW, Germany), and aliquots were snap‐frozen in liquid nitrogen for storage at −80 °C.

### Isothermal titration calorimetry

The binding of GDP, GTP, ppGpp and pppGpp to (*cp*)SRP54 or (*cp*)FtsY‐NG was assessed using a MicroCal ITC200 instrument (Malvern Panalytical; Kassel, Hessen, Germany). For each measurement, 25 μm of protein and 1 mm of ligand were diluted in buffer C and loaded into the cell and syringe, respectively. The experiments were conducted at 25 °C with a stirring rate of 750 rpm, involving one injection of 0.4 μL followed by 12 injections of 3 μL and 150 s of spacing between them. The raw data were processed using the MicroCal PEAQ‐ITC Analysis Software v1.41 (Malvern Panalytical) employing a One Set of Sites model. All the nucleotides used for ITC and other experiments were purchased from Jena Bioscience. The thermodynamic values of these analyses are presented in Table S1.

### Complex formation analysis by analytical size‐exclusion chromatography

**Fig. 1:**
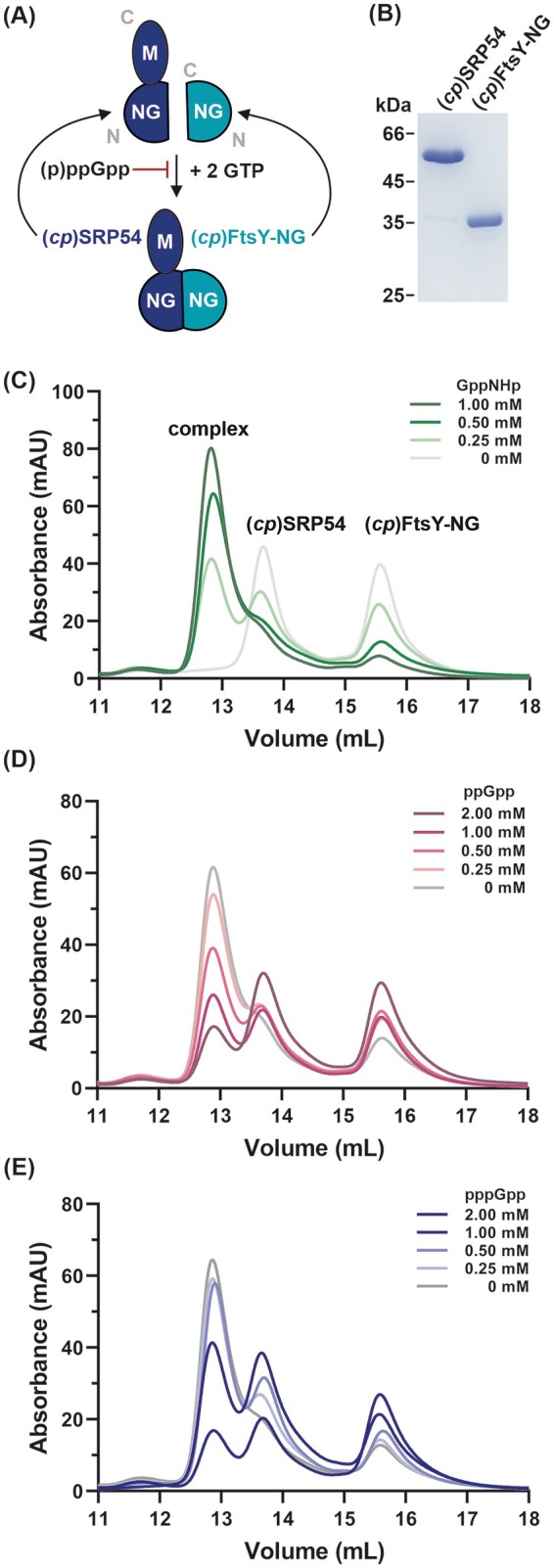
(p)ppGpp disrupts the association of (*cp*)SRP54 to (*cp*)FtsY‐NG. (A) Working model of the two plastidial GTPases (*cp*)SRP54 and (*cp*)FtsY‐NG. (B) SDS/PAGE of recombinantly produced His‐tagged proteins after a two‐step purification procedure, including affinity‐ and size‐exclusion chromatography (SEC). The formation of a complex between equimolar concentrations of (*cp*)SRP54 and (*cp*)FtsY‐NG was assayed in the presence of increased levels of the nonhydrolysable GTP analogue GppNHp. (C) Complex formation between (*cp*)SRP54 and (*cp*)FtsY‐NG was tested in the presence of 0.5 mm GppNHp and increasing concentrations of (D) ppGpp or (E) pppGpp.

(*cp*)SRP54 and (*cp*)FtsY‐NG were diluted in a reaction volume of 150 μL to 100 μm in buffer C in the presence of the previously mentioned nucleotides at the indicated concentrations (Fig. 1C–E). Samples were incubated for 30 min at room temperature, centrifuged (10 000 ***g*** for 5 min, 4 °C) and 100 μL of the supernatant was then loaded on to a Superdex™ 200 Increase 10/300 GL column (Cytiva) for its analysis.

### High‐performance liquid chromatography (HPLC)

To assess the GTPase activity of (*cp*)SRP54, (*cp*)FtsY‐NG and their complex, enzymatic reactions containing 10 μm of each protein, 500 μm of GTP and increasing concentrations of ppGpp or pppGpp were incubated at 37 °C for 5 or 30 min in Buffer D [25 mm HEPES‐K, pH 7.5, 10 mm Mg(OAc)_2_, 300 mm K(OAc), 1 mm DTT and 2.5% (v/v) glycerol]. Samples were taken immediately after initiating the reaction with GTP (*t*_0m_) and at the end of the incubation period (*t*_5m_ or *t*_30m_). Samples were quenched by adding chloroform, vortexing for 5 s and heating at 95 °C for 15 s, followed by rapid freezing in liquid nitrogen. After thawing, samples were centrifuged at 13 000 ***g*** for 5 min at 4 °C, and the aqueous phase was analysed via HPLC using the Agilent 1260 Infinity system (Agilent; Santa Clara, CA, USA) equipped with a Metrosep A Supp 5 – 150/4.0 column (Metrohm, Filderstadt, BW, Germany). Nucleotides were eluted isocratically at a flow rate of 0.6 mL·min^−1^ in Buffer E [90 mm (NH4)_2_CO_3_, pH 9.25] and detected at 260 nm. GDP and GTP solutions served as standards to identify the retention time of these nucleotides in our experimental samples. GTP consumption was then quantified by comparing the peak area corresponding to GTP at *t*_0m_ to that of *t*_5m_ or *t*_30m_. All enzymatic reactions were done in triplicates.

### Crystallization and structure determination

Crystals of (*cp*)FtsY‐NG were grown by the hanging drop vapour diffusion method at 20 °C. The protein to well solution ratio was 1 μL to 1 μL, the well containing 1 mL solution. Protein solutions of 3 mm were incubated with 9 mm (final concentration) pppGpp and ppGpp for 10 min at room temperature. The final crystallization conditions used for structure determination were 100 mm Tris pH 8.5 and 25% (w/v) PEG1000. Prior to data collection, crystals were flash‐frozen in liquid nitrogen using a cryo‐solution that consisted of mother‐liquor supplemented with 20% (v/v) glycerol. Data were collected under cryogenic conditions at the European Synchrotron Radiation Facility (Grenoble, France81). MxCube2 and MxCube3 were used for data collection (https://github.com/mxcube). Data were processed with XDS (version January 31, 2020) and scaled with XSCALE82 [42]. All structures were determined by molecular replacement with PHASER83 [43], manually built in coot84 (Coot Version 0.9.4.1) [44] and refined with phenix85 (Phenix Version 1.17.1‐3660 and 1.19) [45]. A structure of *A. thaliana* (*cp*)FtsY was already known and has been used as a model for molecular replacement (PDB: 2OG2) Figures were prepared with ucsf chimerax version 1.8 [46, 47, 48].

## Results

### (p)ppGpp binds (*cp*)SRP54 and (*cp*)FtsY‐NG with affinities comparable to GDP and GTP

The (*cp*)SRP54 comprises two key domains: the N‐terminal GTPase (NG) domain, responsible for GTP binding and hydrolysis, followed by the methionine‐rich (M) domain, which mediates interactions with signal sequences and (*cp*)SRP43 (Fig. 1A) [9, 49, 50, 51]. Similarly, (*cp*)FtsY features an NG domain that facilitates its interaction with (*cp*)SRP54 (Fig. 1A). Previous studies have shown that the interaction between the NG domains of (*cp*)SRP54 and (*cp*)FtsY is GTP‐dependent [52].

In bacteria, the alarmones (p)ppGpp can bind to the GTPases of Ffh (54 homologue; the bacterial SRP54) and FtsY, acting as competitive inhibitors of Ffh:FtsY complex formation [40]. To investigate whether a similar mechanism applies to the (*cp*)SRP system, we used isothermal titration calorimetry (ITC) to assess the binding of ppGpp and pppGpp, along with their GDP and GTP counterparts. Determination of the binding affinity of ppGpp and pppGpp for (*cp*)SRP54 revealed dissociation constants of 32.5 ± 5.4 μm and 47.8 ± 3.5 μm, respectively (Table 1, Table S1, Fig. S1A,B). The binding affinities for the alarmones are similar to their corresponding counterparts as evidenced by a *K*_d_ value of 23.5 ± 2.3 μm for GDP and 51.8 ± 3.5 μm for GTP (Table 1, Table S1, Fig. S1C,D). Next, we confirmed the binding of ppGpp and pppGpp to (*cp*)FtsY‐NG, displaying dissociation constants of 11.9 ± 2.4 μm and 27.6 ± 3.6 μm, respectively (Table 1, Table S1, Fig. S1E,F). As is the case for (*cp*)SRP54, the *K*_d_ values for GDP, 7.71 ± 0.4 μm and GTP, 11.9 ± 2.4 μm, are comparable to ppGpp and pppGpp, respectively (Table 1, Table S1, Fig. S1G,H). ppGpp and pppGpp bind (*cp*)SRP54 and (*cp*)FtsY‐NG with affinities similar to GDP and GTP, making them putative competitive inhibitors of both SRP‐GTPases.

**Table 1:** Isothermal titration calorimetry (ITC) dissociation constants.

Experiment		*K*_d_ (μm)
(*cp*)SRP54	GDP	23.50 ± 2.29
	GTP	51.80 ± 3.54
	ppGpp	32.50 ± 5.38
	pppGpp	47.80 ± 3.52
(*cp*)FtsY‐NG	GDP	7.71 ± 0.40
	GTP	11.90 ± 2.44
	ppGpp	7.36 ± 1.46
	pppGpp	27.6 ± 3.64

### (p)ppGpp hinders formation of (*cp*)SRP54:FtsY‐NG complex

Next, we examined whether (p)ppGpp could inhibit GTP‐dependent complex formation between (*cp*)SRP54 and (*cp*)FtsY, similar to what has been observed in the bacterial system. Firstly, we verified the complex between the two recombinantly produced GTPases (*cp*)SRP54 and the NG domain of (*cp*)FtsY ((*cp*)FtsY‐NG) from *Arabidopsis thaliana* (Fig. 1B) by incubating equal concentrations of the proteins in the presence of GppNHp, a nonhydrolysable GTP analogue (Fig. 1C). Using analytical size‐exclusion chromatography (SEC), we clearly visualized the complex between (*cp*)SRP54 and (*cp*)FtsY‐NG at increasing concentrations of GppNHp (Fig. 1C). After preincubating both proteins with 0.5 mm of GppNHp, we titrated increasing concentrations of ppGpp and repeated the chromatographic setup, showing that ppGpp indeed disrupts the complex (Fig. 1D). The same conclusions were reached after pppGpp was titrated into the reaction albeit no significant inhibition was observed at equimolar concentrations of GppNHp:pppGpp (Fig. 1E). Overall, we confirm that the binding of (p)ppGpp to (*cp*)SRP and/or (*cp*)SRP receptor GTPases results in the destabilization of their complex, a mechanistically essential checkpoint in the pathway towards membrane protein insertion.

### (p)ppGpp inhibits the GTPase activities of (*cp*)SRP54 and (*cp*)FtsY‐NG

**Fig. 2:**
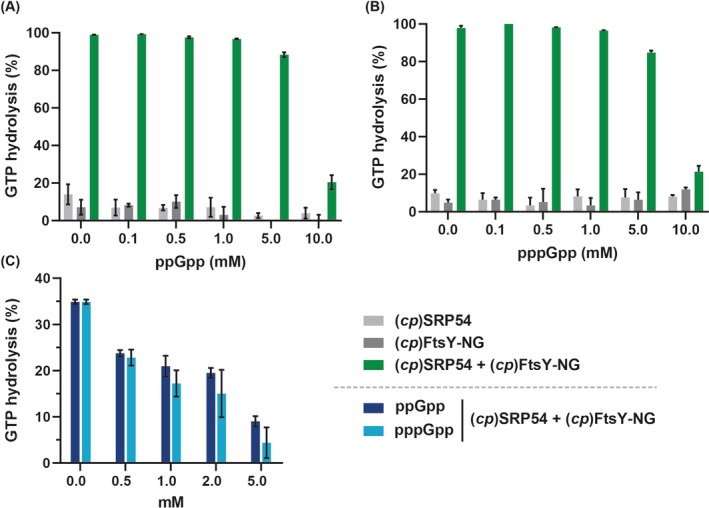
(p)ppGpp reduces GTPase activity (*cp*)SRP54 and (*cp*)FtsY‐NG. GTPase activity of full‐length (*cp*)SRP54 and (*cp*)FtsY‐NG was assayed in the presence of increasing amounts of the competitors ppGpp (A) and pppGpp (B). Enzymatic reactions containing 10 μm of each protein, 500 μm of GTP and increasing concentrations of (p)ppGpp were incubated at 37 °C for 30 or 5 min (C). The data represent mean values (±SD) of *n* = 3 replicates.

To further explore the regulation of (*cp*)SRP54 and (*cp*)FtsY‐NG by (p)ppGpp, we assayed their ability to hydrolyse 500 μm of GTP, both individually and as a complex, at increasing concentrations of the alarmones. The reactions were incubated for 30 min. Consistent with previous reports on the bacterial homologues of (*cp*)SRP54 and (*cp*)FtsY‐NG [40], the individual proteins exhibited low GTPase activity (~ 10% of the available GTP was hydrolysed), which was significantly enhanced when in complex, hydrolysing nearly all available GTP (Fig. 2A,B). For clarity, 100% GTPase activity would correspond to the hydrolysis of all GTP provided in the reaction within the incubation period.

(p)ppGpp showed a mild inhibition of (*cp*)SRP54:FtsY‐NG starting at equimolar concentrations of the supplied GTP (Fig. 2A,B). At 5 and 10 mm, (p)ppGpp further inhibited the GTPase activity of the complex, decreasing the values to roughly 20% at the highest concentration tested. Given that the complex hydrolysed nearly all GTP within 30 min, we hypothesized that a shorter incubation time might reveal subtler inhibitory effects of (p)ppGpp that could otherwise be overlooked. Hence, we repeated the experiment with a reaction time of 5 min. As expected, the inhibitory effect of (p)ppGpp at equimolar concentrations of GTP was more pronounced at *t*_5m_ than at *t*_30m_, with activity dropping from ~ 35% to ~ 25% (Fig. 2C). Furthermore, the GTPase activity of the complex fell below ~ 10% at 5 mm (p)ppGpp, showcasing the ability of the alarmones to inhibit the enzymes over shorter time frames. No discernible differences were detected between ppGpp and pppGpp. Taken together, our data confirm that (p)ppGpp effectively downregulates the GTPase activity of the chloroplast GTPases (*cp*)SRP54 and (*cp*)FtsY.

### Structural characterization of ppGpp bound to (*cp*)FtsY‐NG

**Fig. 3:**
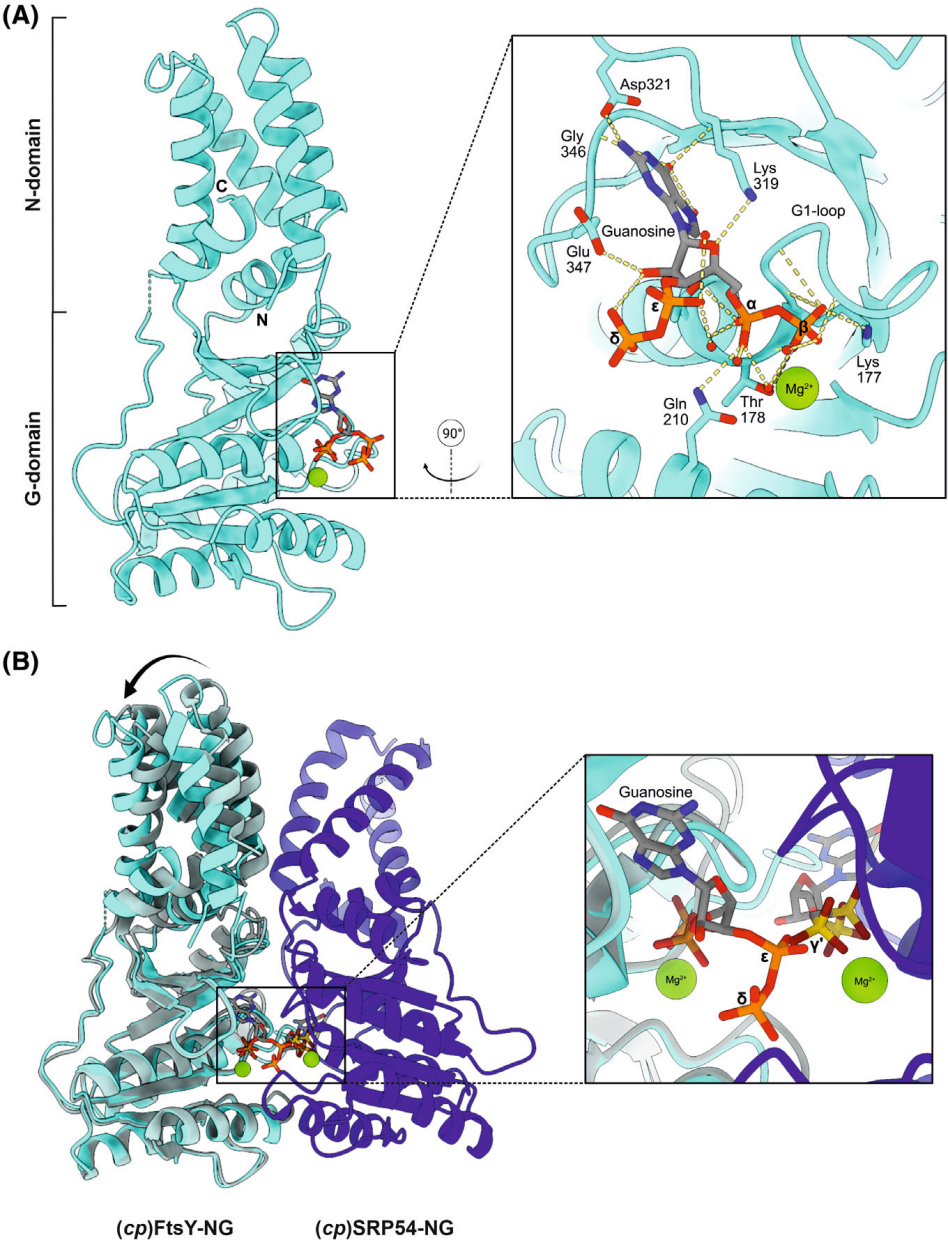
Crystal structure of (*cp*)FtsY‐NG from *Arabidopsis thaliana* bound to ppGpp. (A) Overall ribbon representation of (*cp*)FtsY‐NG in complex with ppGpp (this study) at a resolution of 1.99 Å. Zoom‐in into the nucleotide‐binding site of (*cp*)FtsY‐NG depicting the main residues involved in ppGpp binding. (B) Ribbon diagram of our (*cp*)FtsY‐NG:ppGpp structure superimposed onto the (*cp*)FtsY‐NG:SRP54 heterodimeric structure of Wild & Bange *et al*. (PDB: 5L3R) in complex with GppCp. Superposition analysis resulted in an RMSD of 0.427 Å. A rotation of the N domain towards the G domain of our structure with respect to PDB: 5L3R is marked with an arrow. Close‐up view highlights the steric clash and electrostatic repulsion that would result between the δ‐ and ε‐phosphates of the alarmone and the ɣ‐phosphate of the GTP analogue bound to (*cp*)SRP54. For the structural analysis between these complexes, the GppCp bound to (*cp*)FtsY‐NG of Wild & Bange *et al*. is hidden while the ppGpp of our structure is shown. Colour coding: (*cp*)FtsY‐NG:ppGpp (this study), cyan; (*cp*)FtsY‐NG (Wild & Bange *et al*.), grey, and (*cp*)SRP54:GppCp (Wild & Bange *et al*.), dark blue.

To investigate alarmone binding to chloroplast SRP‐GTPases, we attempted to crystallize (*cp*)SRP54 and (*cp*)FtsY‐NG with ppGpp and pppGpp. Although crystallization of (*cp*)SRP54 with either ppGpp or pppGpp was unsuccessful, we successfully obtained diffracting crystals of (*cp*)FtsY‐NG in the presence of ppGpp. We were able to solve the crystal structure of the truncated form of (*cp*)FtsY from *A. thaliana* (lacking the first 64 amino acids, including the 41‐long transit peptide sequence for import into the chloroplast and the membrane‐binding amphipathic helix) in complex with magnesium and ppGpp by molecular replacement at 1.99 Å resolution (Fig. 3A, Table 2).

**Table 2:** Data collection and refinement statistics for (*cp*)FtsY‐NG:ppGpp.

Parameters	Data^a^
Space group	C 1 2 1
Cell dimensions	
a, b, c (Å)	138.28, 37.07, 59.82
α, β, γ (°)	90, 110.628, 90
Wavelength (Å)	0.976253
Resolution (Å)	35.64–1.99 (2.061–1.99)
Unique reflections	19 770 (1978)
Completeness (%)	99.63 (99.50)
Multiplicity	6.3 (5.5)
*I*/*σI*	14.86 (2.95)
*R*_merge_	0.1176 (0.5475)
*R*_pim_	0.05077 (0.2551)
CC_1/2_	0.996 (0.917)
Refinement	
Reflections	19 757 (1975)
Reflections (*R*_free_)	984 (97)
*R*_work_	0.2194 (0.3331)
*R*_free_	0.2759 (0.4011)
No. of atoms	2363
Macromolecule	2224
Ligand	37
Water	102
RMSDs	
Bond lengths (Å)	0.009
Bond angles (°)	1.15
Ramachandran (%)	
Favoured	97.25
Allowed	2.41
Outliers	0.34
Rotamer outliers	0.83

^a^ Statistics for the highest‐resolution shell are shown in parentheses.

The structure of (*cp*)FtsY‐NG shows residues 65–366 and displays the typical domain structure of SRP‐GTPases consisting of two segments: the N‐terminal N domain comprising four ⍺‐helices (⍺N1 to ⍺N4; residues 65–150), and the C‐terminal G domain (residues 153–366) containing the G elements (G1‐G5) and the typical I‐box (⍺‐β‐⍺) insertion (residues 205–247). The first 23 residues of the mature protein and residues 151–152 are disordered. ppGpp fits precisely within the canonical guanosine nucleotide‐binding site, occupying the space where GTP or GDP would be present. The G1 element or the P‐loop (GVNGGGKT in our structure) forms an anion hole coordinating the ⍺‐ and β‐phosphates of ppGpp; the G2 and G3 elements, DTFR and DTSG, respectively, also contribute to this arrangement. The G4 element (TKLD) provides the coordination and recognition of the guanine moiety mainly through Asp321 and Lys319; the latter also stabilizing the alarmone's ribose (Fig. 3A). Additionally, the G5 element (GVGE) provides a similar coordination to the guanine and ribose moieties through Gly346 and Glu347 (Fig. 3A). Interestingly, the δ‐ and ε‐phosphates covalently bound to the 3′OH of the ribose face outwards of the GTPase's active site without any major coordination (Fig. 3A). In summary, our structural analysis of (*cp*)FtsY‐NG in complex with ppGpp reveals that the alarmone interacts similarly to GDP. This indicates that the additional pyrophosphate group at the 2′‐hydroxyl of its ribose serves as the key inhibitory element of (p)ppGpp.

### Molecular mechanism of ppGpp‐dependent inhibition of the (*cp*)SRP54:FtsY complex

Previous reports have shown that the heterodimeric complex between (*cp*)SRP54 and (*cp*)FtsY, and to that extent also the SRP system from prokaryotes, eukaryotes and archaea, enforces a tightly coordinated catalytic centre [53]. In this centre, each GTPase establishes the GTP molecule of its counterpart via hydrogen bonding between the 3′OH ribose of one molecule and the ɣ‐phosphate of the other [53, 54, 55, 56, 57]. When ppGpp is bound to (*cp*)FtsY, however, the formation of the (*cp*)SRP54:FtsY complex is hindered due to a steric clash and electrostatic repulsion from the negatively charged δ‐ and ε‐phosphates of the alarmone, ultimately disrupting the SRP heterodimer. This is evident when the P‐loop region (residues 201–301) of our (*cp*)FtsY‐NG structure is superimposed onto that of Wild & Bange *et al*. (PDB: 5L3R), resulting in a root mean square deviation (RMSD) of 0.427 Å over 510 atoms, with (*cp*)SRP54 from their structure included for visualization purposes (Fig. 3B). Moreover, we see a rotation of the N domain when ppGpp is bound in comparison with the GppCp‐bound structure, suggesting a more rigid configuration between the N and G domain that could possibly contribute to a less‐prone‐to‐dimerize conformation. All in all, our structural observations provide clear insights into how the alarmone ppGpp, and most likely also pppGpp, would hinder the (*cp*)SRP targeting complex by impeding the GTPases to interact with each other.

## Discussion

Although ppGpp has been initially identified as a direct binder of plastidial RNA polymerase in *Spinacia oleracea*, adenylosuccinate synthetases in *Oryza sativa* and guanylate kinases in *Oryza sativa*, *Pisum sativum* and *Arabidopsis thaliana* [37, 38, 39], its precise role in chloroplasts remains poorly understood. In bacteria, (p)ppGpp binds to Ffh and FtsY, disrupting their interaction and impairing SRP‐dependent membrane protein targeting [40]. Given the presence of bacterial SRP homologues in chloroplasts, we explored whether (p)ppGpp exerts similar regulatory effects on the chloroplast SRP and SRP receptor GTPases.

### The inhibition of the (*cp*)SRP by (p)ppGpp shows mechanistic and structural similarities but also differences from its bacterial counterpart

Using ICT, we confirmed that ppGpp and pppGpp bind to (*cp*)SRP54 and (*cp*)FtsY‐NG with affinities akin to their bacterial counterparts [40]. Biochemical analyses suggest that apo‐(*cp*)FtsY adopts a preorganized, closed state that facilitates (*cp*)SRP54 association [15], although direct evidence is lacking, while molecular dynamic simulations demonstrate that GTP is essential for stable complex formation, inducing conformational changes in (*cp*)FtsY that promote interaction with (*cp*)SRP54 [58]. Consistent with these findings, we observed complex formation only in the presence of GppNHp, while (p)ppGpp competes with it to disrupt this interaction. This inhibition mechanism mirrors observations in bacteria [40], suggesting conserved regulatory strategies to modulate the SRP in both systems. However, our initial GTP hydrolysis assays showed minimal inhibition by (p)ppGpp except at high concentrations, unlike bacterial SRP‐GTPases [40]. In the bacterial assays, approximately 30% GTPase activity remained after 1 h, allowing (p)ppGpp to act, whereas our assays showed near‐complete GTP hydrolysis within 30 min, potentially masking inhibition. The rapid nucleotide exchange characteristic of SRP‐type GTPases [7, 59, 60] likely facilitated GTP replacement of bound (p)ppGpp during longer incubation. Reducing the reaction time to 5 min revealed stronger inhibitory effects, even at equimolar GTP concentrations.

Given the lower concentrations of ppGpp in chloroplasts compared with bacteria, (p)ppGpp may regulate the chloroplast SRP pathway by targeting earlier steps rather than directly inhibiting GTPase activity. This hypothesis aligns with findings by Nguyen *et al*. [61], who explored the relative importance of GTPase activation in the chloroplast SRP system compared with its cytosolic counterpart. One key aspect to consider is the extent to which SRP complex assembly and GTPase activation are coupled, and whether this coupling differs between bacteria and chloroplasts. In bacteria, mutations that impair the GTPase activity of Ffh and FtsY do not result in a significant disruption of their complex [62]. By contrast, similar mutations in (*cp*)SRP54 and (*cp*)FtsY severely compromise complex assembly, but have minimal effects in the targeting of LHCP. This suggests that, in chloroplasts, complex formation and GTPase activation are closely integrated, while in bacteria, these two steps occur separately [61]. The uncoupling of these steps in bacteria may provide an additional fidelity checkpoint, advantageous for discriminating between a broader range of substrates [63, 64]. In contrast, the chloroplast SRP system handles a more homogenous substrate pool, primarily targeting the highly conserved Light‐Harvesting Complex protein superfamily [65, 66, 67] aided by the substrate recognition features of (*cp*)SRP43 [68, 69].

Interestingly, our complex disruption experiments revealed distinct differences between ppGpp and pppGpp, even at lower concentrations, which were not reflected in the GTPase inhibition assays. This suggests that the alarmones may exert additional effects beyond complex disruption, potentially inhibiting GTPase activity directly. This possibility highlights a dual regulatory role for (p)ppGpp in the chloroplast SRP pathway, with complex disruption and GTPase inhibition acting in concert to fine‐tune SRP function and with ppGpp and pppGpp potentially affecting this to different extents. Consequently, (p)ppGpp may regulate the chloroplast SRP pathway differently than its bacterial counterpart. In doing so, (p)ppGpp might also limit the availability of GTP‐bound (*cp*)FtsY, preventing its association with (*cp*)SRP54 and restricting (*cp*)SRP turnover. This mechanism aligns with increased ppGpp levels during the night [70, 71], when chloroplast activity and protein translocation demands are lower [72, 73].

Structurally, the binding of ppGpp to the NG domain of (*cp*)FtsY and its resulting inhibition of the complex with (*cp*)SRP54 closely resemble the mechanism observed in its bacterial counterparts [40]. The negatively charged δ‐ and ε‐phosphates of the alarmone disrupt the coordination of the two GTP molecules required to stabilize the (*cp*)SRP54:FtsY heterodimer. Uniquely, our findings suggest that the binding of ppGpp induces a slight rearrangement of the NG domain, favouring a more open conformation, which may be characteristic of the chloroplast SRP FtsY receptor. In contrast, GTP‐bound (*cp*)FtsY adopts a closed conformation, mediated by NG domain reorientation, that is required for its efficient association to (*cp*)SRP54 [58]. The structural rearrangement caused by ppGpp appears to oppose this effect, further hindering complex formation. It is likely that pppGpp binds and inhibits (*cp*)FtsY in a similar manner to ppGpp; however, whether it also induces the less‐prone to associate open conformation of the NG domain requires further structural research.

### Is this a physiologically relevant regulation?

The discovery of ppGpp in plants marks a significant advancement in our understanding of plant physiology and stress responses. Studies reveal that ppGpp accumulation profoundly impacts chloroplast function, photosynthesis and plant growth. During the night, chloroplast ppGpp levels have been estimated at 3 μm [30]. It has been shown that ppGpp accumulation correlates with GTP depletion under specific conditions, particularly during nitrogen deprivation [31, 32, 74]. This phenomenon mirrors observations in bacteria, where GTP levels decrease as ppGpp levels rise (reviewed in [75]), enhancing the competitive advantage of ppGpp. This is attributed to ppGpp's dual role in utilizing GTP as a substrate for its synthesis and inhibiting enzymes involved in chloroplast GTP biosynthesis, such as guanylate kinase [39]. Furthermore, the regulatory range of (p)ppGpp in bacteria spans dissociation constants from 0.01 μm to approximately 1 mm [16], suggesting that chloroplast ppGpp levels could modulate multiple targets. For instance, ppGpp inhibits plastid‐encoded plastid RNA polymerase, while sparing the nuclear‐encoded form, highlighting the alarmone's subcellular specificity [37].

In additional to transcriptional control, ppGpp also disrupts mRNA translation in chloroplast extracts [76]. Interestingly, the majority of chloroplast translation factors (IF2, EFG, EF‐TU, EF‐Ts) retain bacterial‐like features following endosymbiosis and the evolution of plants [77, 78, 79, 80, 81, 82]. Nevertheless, while some translation‐related targets of ppGpp have been proposed, they remain to be directly characterized [76]. Similar to bacteria, ppGpp may regulate chloroplast metabolism through multiple layers, including transcription, translation and the SRP‐dependent protein targeting pathway. A key question arises: why would ppGpp affect (*cp*)SRP‐GTPases, which exhibit weaker dissociation constants? As shown by Vinogradova *et al*. [83], ribosome‐bound bacterial IF2's regulation by ppGpp varies depending on the mRNA template. It is plausible that (p)ppGpp specifically regulates the SRP‐dependent insertion of certain proteins into the thylakoid membrane by hindering the binding of cargo‐loaded (*cp*)SRP to its receptor. This proposed mechanism aligns with the role of ppGpp in downregulating photosynthesis, as it may act by selectively reducing the insertion of components into the photosystem [34], thereby compromising the assembly of photosynthetic complexes. This regulation could confer an advantage by maintaining a pool of translocation‐ready proteins, which would otherwise not be subject to transcriptional or translational control. When ppGpp levels decrease, potentially during the diurnal cycle, these proteins could be rapidly mobilized, restoring the photosynthetic capacities of chloroplasts in response to the plant's metabolic needs.

## Author contributions

PB and GB conceived and supervised the study. VZ performed the enzyme assays, ITC experiments and SEC analysis. PW, PAP and JT cloned plasmids and purified proteins. LC and PB determined the crystal structure. FW provided reagents and relevant discussion. VZ, PB and GB wrote the manuscript. All authors commented on the manuscript.

### Peer review

The peer review history for this article is available at https://www.webofscience.com/api/gateway/wos/peer‐review/10.1002/1873‐3468.70008.

## Supporting information


**Fig. S1.** Ligand binding of (*cp*)SRP54 and of (*cp*)FtsY‐NG determined by ITC.
**Table S1**. Isothermal titration calorimetry (ITC) parameters.

## Data Availability

The atomic coordinates and structure factors have been deposited in the Protein Data Bank with the accession code 8RIX.
