# Supplementary material for: Structural and mechanistic basis for the regulation of the chloroplast signal recognition particle by (p)ppGpp
Source: FEBS Lett. 2025 Feb 11;599(10):1373–85. doi: 10.1002/1873-3468.70008 (PMC12104861; doi:10.1002/1873-3468.70008)
Supplement: Supplementary file 1 — Fig. S1. Ligand binding of (cp)SRP54 and of (cp)FtsY‐NG determined by ITC. Table S1. Isothermal titration calorimetry (ITC) parameters. [file FEB2-599-1373-s001.pdf]

*Supporting Information for:*

# Structural and mechanistic basis for the regulation of the chloroplast signal recognition particle by (p)ppGpp

Victor Zegarra[1,2], Paul Weiland[1,3], Pauline Plitzko[1,2], Julia Thiery[1], Laura Czech[1], Felix Willmund[1,4], Patricia Bedrunka[1,2*] and Gert Bange[1,2,5*]

[1]Center for Synthetic Microbiology (SYNMIKRO), Philipps University Marburg, Germany

[2]Department of Chemistry, Philipps-University Marburg, Marburg, Germany

[3]Center for Tumor Biology and Immunology, Department of Medicine, Philipps-University Marburg, Marburg, Germany

[4]Department of Biology, Philipps-University Marburg, Marburg, Germany

[5]Max Planck Institute for Terrestrial Microbiology, Marburg, Germany

[*]Correspondence: patricia.bedrunka@synmikro.uni-marburg.de, gert.bange@synmikro.uni-marburg.de

**The file contains:**

Supplementary Fig. S1

Supplementary Table S1

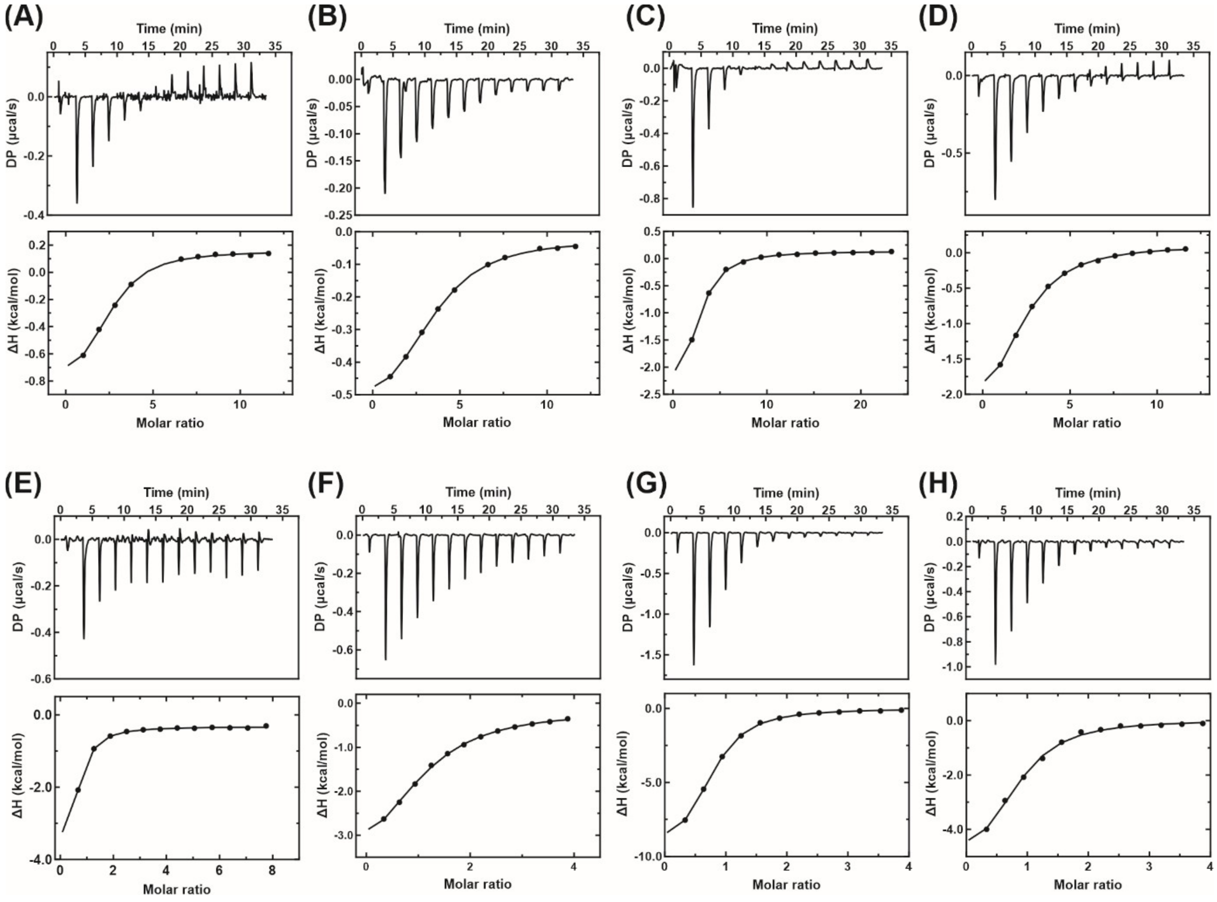


**Fig. S1.** Ligand binding of (*cp*)SRP54 and of (*cp*)FtsY-NG determined by ITC. Purified (*cp*)SRP54 (A-D) and (*cp*)FtsY-NG (E-H) were titrated with ppGpp (A,E), pppGpp (B,F), GDP (C,G) and GTP (D,H) and the binding was assessed via ITC, respectively. The upper part of each panel shows the raw injection heats (µcal $s^{-1}$). The lower part of each panel displays the corresponding specific binding isotherms (Kcal $mol^{-1}$ of injectant) plotted against the molar ratio.

| Experiment | $K_d$ | ΔH | -TΔS | ΔG | N |
|---|---|---|---|---|---|

| | | (µM) | (kcal mol$^{-1}$) | (kcal mol$^{-1}$) | (kcal) | (sites) |
|---|---|---|---|---|---|---|
| **(*cp*)SRP54** | GDP | 23.50 ± 2.29 | -3.27 | -3.05 | -6.32 | 2.07 |
| | GTP | 51.80 ± 3.54 | -2.87 | -2.98 | -5.85 | 2.18 |
| | ppGpp | 32.50 ± 5.38 | -1.09 | -5.03 | -6.12 | 2.34 |
| | pppGpp | 47.80 ± 3.52 | -0.59 | -5.31 | -5.90 | 3.36 |
| **(*cp*)FtsY-NG** | GDP | 7.71 ± 0.40 | -5.30 | 3.38 | -6.98 | 0.69 |
| | GTP | 11.90 ± 2.44 | -5.84 | -0.88 | -6.72 | 0.78 |
| | ppGpp | 7.36 ± 1.46 | -5.30 | -1.71 | -7.00 | 0.40 |
| | pppGpp | 27.6 ± 3.64 | -4.11 | -2.11 | -6.22 | 1.06 |

**Table S1.** Isothermal titration calorimetry (ITC) parameters. Thermodynamic values determined from the ITC experiments.
